# Adventitial Microcirculation Is a Major Target of SARS-CoV-2-Mediated Vascular Inflammation

**DOI:** 10.3390/biom11071063

**Published:** 2021-07-20

**Authors:** Francesco Vasuri, Carmen Ciavarella, Salvatore Collura, Chiara Mascoli, Sabrina Valente, Alessio Degiovanni, Mauro Gargiulo, Miriam Capri, Gianandrea Pasquinelli

**Affiliations:** 1 Pathology Unit, IRCCS, Azienda Ospedaliero-Universitaria di Bologna, 40138 Bologna, Italy; francesco.vasuri@aosp.bo.it (F.V.); alessio.degiovanni@aosp.bo.it (A.D.); gianandr.pasquinelli@unibo.it (G.P.); 2 Department of Experimental, Diagnostic and Specialty Medicine (DIMES), University of Bologna, 40138 Bologna, Italy; salvatore.collura4@unibo.it (S.C.); sabrina.valente2@unibo.it (S.V.); miriam.capri@unibo.it (M.C.); 3 Vascular Surgery Unit, IRCCS, Azienda Ospedaliero-Universitaria di Bologna, 40138 Bologna, Italy; chiara.mascoli@aosp.bo.it (C.M.); mauro.gargiulo2@unibo.it (M.G.); 4 Interdepartmental Center “Alma Mater Research Institute on Global Challenges and Climate Change (Alma Climate)”, University of Bologna, 40126 Bologna, Italy

**Keywords:** artery disease, COVID-19, immunohistochemistry, inflammation, miRNA, IL-6, SARS-CoV-2, transmission electron microscopy

## Abstract

We report the case of a 77-year-old woman affected by coronavirus disease-19 (COVID-19) who developed an occlusive arterial disease of the lower limb requiring a left leg amputation. We studied the mechanisms of vascular damage by SARS-CoV-2 by means of a comprehensive multi-technique in situ analysis on the diseased popliteal arterial district, including immunohistochemistry (IHC), transmission electron microscopy (TEM) and miRNA analysis. At histological analyses, we observed a lymphocytic inflammatory infiltrate, oedema and endothelialitis of adventitial vasa vasorum while the media was normal and the intima had only minor changes. The vasa vasorum expressed the ACE2 receptor and factor VIII; compared with the controls, VEGFR2 staining was reduced. TEM analyses showed endothelial injury and numerous Weibel–Palade bodies in the cytoplasm. No coronavirus particle was seen. IL-6 protein and mRNA, together with miR-155-5p and miRs-27a-5p, which can target IL-6, were significantly increased compared with that in the controls. Our case report suggests an involvement of adventitial artery microcirculation by inflammation in the course of COVID-19. Without evident signs of current infection by SARS-CoV-2, endothelial cells show a spectrum of structural and functional alterations that can fuel the cardiovascular complications observed in people infected with SARS-CoV-2.

## 1. Introduction

The coronavirus disease-2019 (COVID-19), caused by the coronavirus SARS-CoV-2, was declared a pandemic by the World Health Organization on March 2020 [1], representing a global threat to health. COVID-19 is associated with respiratory failure due to pulmonary viral pneumonia and cardiovascular disease. Indeed, the plentiful literature on the topic has demonstrated that, unlike other respiratory viruses, COVID-19 is able to trigger cardiovascular complications, including myocarditis, arrhythmias, cardiac injury, heart failure, venous thromboembolism and disseminated intravascular coagulation [2,3]. A 20% incidence of venous thromboembolism at admission was reported at a single center study [4].

The vascular trophism of SARS-CoV-2 is related to the almost ubiquitous angiotensin-converting enzyme 2 (ACE2) receptor present on host cells that the virus uses as an entryway for infection [3]. The ACE2 receptor protein is expressed mostly by the alveolar epithelial cells of the lung, the intestinal enterocytes, as well as by the arterial and venous endothelium [5]. According to recent reports, ACE2 receptor hyperexpression could lead to a more rapid microvascular dysfunction and damage [6], responsible for heart failure, acute coronary syndrome and other potentially fatal multi-organ complications [7].

Here, we report the case of a woman affected by COVID-19 who developed an occlusive arterial disease of the lower limb, characterized by a marked inflammatory status and endothelial activation. In order to delve into the potential mechanisms of vascular damage by SARS-CoV-2, we carried out a comprehensive multi-technique in situ analysis on the diseased vascular district.

## 2. Case Report

The patient was a 77-year-old woman with hypertensive heart disease and early left ventricular dysfunction, 38% ejection fraction and moderate mitral insufficiency. Comorbidities included overweight, type II diabetes, carotid atheromasic disease with bilateral asymptomatic stenosis of 50% (according to the ECTS classification) [8] and a chronic trophic lesion of the right foot, secondary to traumatic fracture treated with rigid osteosynthesis; the trophic lesion was stage V according to the Rutherford classification [9] and grade 2/stage C according to the Texas Wound Classification System [10].

On 19 March 2020, the patient was admitted to the emergency room for hyperpyrexia; a trophic lesion on the left foot was observed at physical examination. She was hospitalized in the geriatric unit, where an ultrasound exam of the distal left lower limb was performed. A deep venous thrombosis of the left soleal vein, a steno-obstruction of the left superficial femoral artery and an occlusion of the ipsilateral posterior tibial artery were found. Anticoagulation with low-molecular-weight heparin at therapeutic dose (enoxaparin 4000 U for 2 days) was started. A qualitative molecular test (Reverse Transcription-Polymerase Chain Reaction (RT-PCR)) on oropharyngeal swabs from the patient was found to be positive for SARS-CoV-2. Nevertheless, due to the worsening of the left foot trophic lesion with signs of infection (grade 2/stage D according to the Texas Wound Classification System [10]), on 26 March, she underwent endovascular angioplasty of the superficial femoral artery, popliteal artery, and first and second ray resection. After surgery, the patient went to the cardiac intensive care unit for 24 h, and after that, she was moved to a dedicated COVID ward, where she started therapy with azithromycin, hydroxychloroquine and tocilizumab, according the regional guidelines for COVID-19 infection. During recovery, she developed a SARS-CoV-2-related interstitial pneumonia with bilateral “ground-glass” features and some consolidation areas, viewed using high-resolution computed tomography (HRCT). The foot lesion worsened dramatically with an increase in inflammation index (stage VI according the Rutherford Classification [9], grade 3/stage D according the Texas Wound Classification System [10]): for this reason, on 4 April, the patient was subjected to new surgery for left leg amputation with ipsilateral knee sparing.

The patient was ASA IV (https://www.asahq.org/standards-and-guidelines/asa-physical-status-classification-system; accessed on 13 December 2020) and required oxygen therapy; she was then operated in an urgent setting under locoregional anesthesia in order to avoid intubation. The tissues of the foot appeared to be severely infected and damaged enough to make it difficult to identify the different anatomical structures. The leg amputation was performed just below the knee, where the tissues were macroscopically viable and perfused. Fresh tissue from the anterior and posterior tibial arteries was collected from the amputated limb for histological analysis.

In the postoperative period, due to the worsening of her respiratory condition and hyperpyrexia, high-flow oxygen therapy was required, without the need for intensive care. In order to reduce pulmonary inflammation, corticosteroid therapy was administered, together with antibiotic therapy with piperacillin, tazobactam and teicoplanin, with progressive normalization of inflammation indexes within two weeks. Her glycemic value was poorly controlled, and consequently, an increase in insulin therapy was needed. The respiratory condition progressively improved and the need for oxygen therapy decreased, but the healing of surgical wounds was difficult, requiring medication three times a week. Nasopharyngeal swabs for the detection of SARS-CoV-2 were performed on 28 and 29 April, with negative results. Another nasopharyngeal swab was repeated on 19 May with a positive result.

Due to the surgical wound dehiscence, a new operation of left leg amputation above the knee was performed on 9 June. The patient’s general condition recovered progressively, and on 22 June, she was discharged to a low-intensity care COVID ward and rehabilitation was undertaken.

The patient came back to our attention for clinical evaluation after 9 months of follow-up, on 24 March 2021: she completely recovered with the absence of lesions and/or rest pain. She went back to walking independently thanks to a left lower limb prosthesis.

## 3. Histopathology and Immunohistochemistry

**Figure 1:**
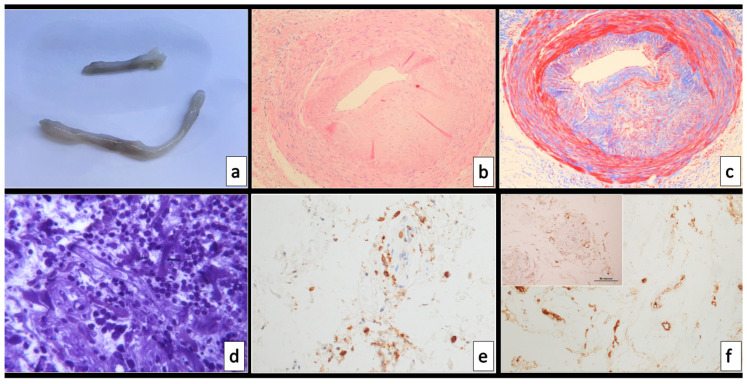
Morphology of the case report. (**a**) Macroscopical appearance of one of the arterial segments examined; hematoxylin–eosin (**b**) and Mallory’s trichrome (**c**) stains: the arterial lumen is reduced by a moderate myointimal thickening, characterized by a scarce cellularity and very mild intimal inflammation. (**d**) Chronic inflammatory infiltrate in the adventitial perivascular tissue, at semi-thin sections, mainly composed by CD3-positive lymphocytes (**e**). Adventitial neoangiogenesis is immunoreactive for FVIII (**f**) and expresses ACE2 receptor as well (insert). Magnification 2× (**b**,**c**) and 20× (**d**–**f**).

The surgical specimens sent to the Pathology Unit included six tibial artery segments from 2 to 5 cm in length with normal structure but with thickened wall and focally reduced lumen (Figure 1a).

Small, randomly selected tissue fragments were sampled, fixed in glutaraldehyde and routinely processed for Transmission Electron Microscopy (TEM), the rest of the tissue was fixed in formalin, embedded in paraffin and routinely processed for histopathological analysis. Two-micrometer-thick sections were stained with hematoxylin–eosin and Picro-Mallory trichrome stains; immunohistochemistry (IHC) was carried out automatically with the automated immunostainer Benchmark^®^ ultra (Ventana Medical Systems, Inc, Roche group, Tucson, AZ, USA) for the following antigens: CD3 (clone 2GV6), CD34 (clone QBEnd-10), factor VIII (FVIII, clone P) and ACE2 receptor (clone EPR44352). IHC for VEGFR (clone D5B1; Cell Signaling; Danvers, MA, USA) and IL-6 (polyclonal, Sigma Aldrich, St. Louis, MO, USA) was manually performed using a non-biotin amplified method (NovoLink Polymer Detection Kit; Leica Biosystems, Wetzlar, Germany) according to the manufacturer instructions. The IHC quantification was performed with ImageJ software and expressed as percentages of positive areas.

In situ hybridization (ISH) for the detection of SARS-CoV-2 antigens was performed with RNAScope^®^ SARS-CoV-2 sense (ACD Biotechne, Minneapolis, MN, USA).

Histological examination demonstrated a significant reduction in the arterial lumen, moderate-to-severe mucoid intimal thickening due to excess of mucopolysaccharides and pools of mucin, with fibrosis and disarray of the medial layer (Figure 1b,c). As demonstrated by CD34 IHC, the endothelial lining was present. No features of atherosclerosis were observed; the inner elastic laminae was intact.

Of note, a mild-to-moderate CD3-positive lymphocytic inflammatory infiltrate was present in the adventitia, associated with focal oedema and endothelialitis of the adventitial vasa vasorum (Figure 1d,e): this was in contrast with the inner media layer, which did not show inflammation.

Some sections showed subacute lesions with fresh and organized thrombus with partial recanalization occluding the arterial lumen. The von Willebrand factor (FVIII) was seen intensely expressed in both surface and microvascular endothelial cells (Figure 1f).

ACE2 IHC showed immunoreactivity in endothelial and some myofibroblastic cells, including adventitial vasa vasorum endothelia (Figure 1f insert).

**Figure 2:**
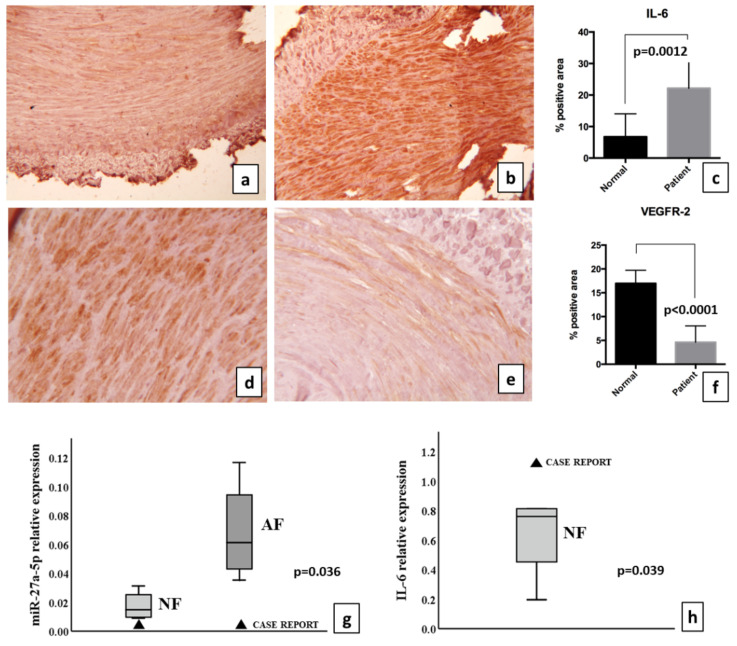
Immunohistochemical and molecular characterization of the case report. Detection of IL-6 in (**a**) normal femoral artery and (**b**) case report; (**c**) quantification of tissue area percentage positive to IL-6, significantly higher in the case report (*p* = 0.0012). Detection of VEGFR-2 in (**d**) the normal femoral artery and (**e**) case report; (**f**) the quantification of the tissue area percentage positive for VEGFR-2, significantly lower in the case report (*p* < 0.0001). (**g**) Real-time RT-PCR analysis of miR-27a-5p; the single value of the case report is compared with normal femoral arteries as a box plot (not significant) and with atherosclerotic femoral arteries as a box plot (*p* = 0.036, *z*-score test). (**h**) mRNA levels of IL-6 in normal femoral arteries and the case report; the single value of the case report is compared with the normal femoral arteries as a box plot (*p* = 0.039, *z*-score test). Triangles represent the case report. Abbreviations: NF: normal femoral artery; AF: atherosclerotic femoral artery.

The quantification of the areas positive for VEGFR-2 and IL-6 revealed a differential expression pattern between normal femoral arteries and case report. Even though IL-6 expression was detected in normal tissue, the percentage of positive areas was significantly higher in the case report (22.1% ± 3.6 in case report, 6.7% ± 2.1 in normal femoral artery; *p* = 0.0012) (Figure 2a–c). Conversely, we found a marked decrease in VEGFR-2 stains in the case report tissue (4.6% ± 1.3 in the pathological tissue, 17% ± 1.12 in the normal femoral arteries; *p* < 0.0001) (Figure 2d–f).

The detection of the virus on tissue by means of ISH was negative.

## 4. Transmission Electron Microscopy

**Figure 3:**
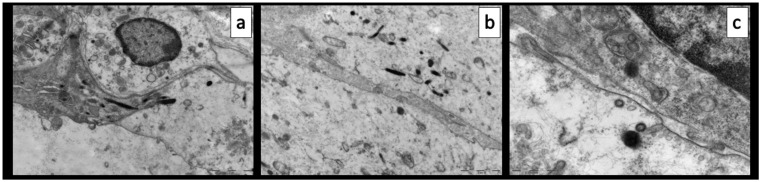
Transmission electron microscopy of the case report. (**a**) Endothelialitis, clear cytoplasm, hypertrophy of Golgi complex and abundance of Weibel–Palade bodies. (**b**) Junctional leakage. (**c**) Virus-like particles with fuzzy proteinaceous cores.

Light microscopy examination of semithin sections confirmed the lympho-monocytic inflammatory infiltrate involving the vasa vasorum in the adventitia (Figure 1d). TEM analysis of these same areas showed endothelialitis with features of endothelial cell activation, e.g., enlarged, euchromatinic nuclei; hypertrophy of Golgi complexes; increased number of Weibel–Palade bodies; and injury, e.g., ballooning with clear cytoplasm and junctional leakage (Figure 3). The presence of Weibel–Palade bodies together with the immunoreactivity for FVIII seems to indicate a pro-thrombotic stimulus in the arterial and capillary endothelial cells, while venular endothelial cells were negative.

Consistent with previous reports, we found increased cytoplasmatic endocytic and round virus-like particles, 120 nm in diameter, with a fuzzy, proteinaceous crown (Figure 3).

## 5. miRNA Analysis

Four normal and four atherosclerotic femoral arteries were compared with the case report. Six different microRNAs (miRs) were detected in all specimens, i.e., miRs-155-5p, -27a-5p and -139-5p and the inflamma-miRs-21-5p, -126-3p and -146a. The first miR-triplet that emerged recently in a previous work on different type of arteries (Collura et al., under review), and the second triplet, recognized as having a role in the inflammatory pathway, were selected [11].

The results showed a significant decrease (*p* = 0.036) in miR-27a-5p expression when the case was compared with femoral atheroma, as shown in Figure 2g. In addition, the case report showed an increased expression of inflamma-miRs-155-5p and -146a (*p* < 0.00001) when compared with normal femoral artery (data not shown). As miR-155-5p and miRs-27a-5p are potentially able to target directly IL-6 mRNA [12], the mRNA was detected as reported in Figure 2h. The increase in mRNA was significant when compared with normal femoral artery (*p* = 0.039). Femoral atheroma did not show IL-6 mRNAs, likely due to the intrinsic characteristics of acellular atherosclerotic plaque (data not shown). The levels of both IL-6 mRNA and protein were increased in the COVID-19 case report. Inflamma-miR-21-5p as well as miR-139-5p were not significantly changed (data not shown).

## 6. Discussion

Endothelial cells are primary targets of SARS-CoV-2 due to the high binding affinity of the viral spike protein towards the ACE2 receptor [3,13]. This explains why organs with high vascularization, such as the lungs and bowels, were described as primary entry routes for SARS coronaviruses long before the current pandemic [5]. Endothelial damage, with the consequent endothelialitis described in course of COVID-19, has always been considered a direct cytotoxic damage caused by SARS-CoV-2 [14]: after entry of the virus to the target cells, the consequent inflammatory response leads to thrombosis and other vascular complications [15].

However, the experience that we report here is likely to indicate that—at least in some cases—the pro-inflammatory and endothelium-activating mechanisms principally concern the endothelial cells of the perivascular microcirculation: in the present case, we observed a higher IHC expression of the ACE2 receptor in the vasa vasorum endothelial cells than in the surface endothelial cells, reaffirming that the inflammation-mediated damage by SARS-CoV-2 might concern the perivascular microcirculation rather than the vessel wall itself.

In the present report, we performed different analyses on the arterial segments from our patient, affected by COVID-19 and occlusive vasculopathy of the lower limbs: histologically, we found a lymphocytic adventitial infiltrate, with activation and injury of the vasa vasorum endothelial cells, which were strongly immunoreactive for FVIII and showed diffuse Weibel–Palade bodies as well as cell junctional leakage at TEM. All of these features are in accordance with the increased pro-thrombotic profile of COVID-19 patients [16]. Of note, serum FVIII is one of the serum markers increased in COVID-19 patients admitted to intensive care units according to a recent report [17].

Here, we also documented an engagement of the endocytic pathway in endothelial cells that should not be misinterpreted [18], as by others [14], as direct evidence of viral infection; the meaning of such activity, that has been associated with COVID-19 viral entry [19], needs to be further explored in relation to the hyperinflammatory syndrome accompanying the clinical course of a cohort of patients infected with COVID-19. In agreement with this view, inhibitors targeting the endocytic pathway, such as chloroquine and hydroxychloroquine, are not effective in the treatment of COVID-19 patients [20] and the ISH of the present case was negative, thus failing to support direct virus infection of small vessel endothelial cells.

Perivascular endothelialitis, associated with IL-6 and relative inflamma-miRs, support the patient’s inflammatory status, which is one of the critical issues currently discussed for COVID-19. Here, we demonstrated the increase in both IL-6 mRNA and protein within the popliteal femoral artery wall, likely mediated by the decrease in miR-27a-5p, one of the potential regulators of IL-6 expression [12]. The presence of IL-6 at the highest level in COVID-19 cases is consistent with the role of IL-6 as a biomarker for the severity of SARS-CoV2 pneumonia [21]. The patient’s inflammatory status is associated with the loss in endothelial cell homeostasis suggested by decreased VEGF receptor expression and endothelial junctional leakage, which can be responsible for facilitating pro-inflammatory changes in the extracellular microenvironment.

The present report is limited to a single-case observation, thus presenting limitations: impossible generalization of the conclusions as well as the danger of overinterpreting the results; moreover, the patient co-morbidities may have influenced the anatomo-clinical evolution of the case. We hereby described a putative mechanism of microvascular damage in people infected with SARS-CoV-2, which is part of the widely recognized spectrum of cardiovascular alterations in the course of COVID-19 disease. The absence of direct virus detection in the patients’ tissue, on one hand, confirms the indirect inflammatory-mediated damage and, on the other hand, makes proving the virus–inflammation–microcirculation interactions difficult.

## 7. Conclusions

Our case report supports the view that adventitial artery microcirculation could be a major target of vascular inflammation in the course of COVID-19; while not appearing to be directly infected by the virus, endothelial cells show a spectrum of structural and functional alterations that are consistent with the cardiovascular complications observed in people infected with SARS-CoV-2.

## Data Availability

All relevant data are available within the manuscript.
